# Single-breath-hold photoacoustic computed tomography of the breast

**DOI:** 10.1038/s41467-018-04576-z

**Published:** 2018-06-15

**Authors:** Li Lin, Peng Hu, Junhui Shi, Catherine M. Appleton, Konstantin Maslov, Lei Li, Ruiying Zhang, Lihong V. Wang

**Affiliations:** 1 Caltech Optical Imaging Laboratory, Andrew and Peggy Cherng Department of Medical Engineering, California Institute of Technology, 1200 East California Boulevard, Pasadena, CA 91125 USA; 2 Department of Biomedical Engineering, Washington University in St. Louis, One Brookings Dr., St. Louis, MO 63130 USA; 3 Breast Imaging Section, Washington University School of Medicine in St. Louis, 510 South Kingshighway Blvd, St. Louis, MO 63108 USA; 4 Caltech Optical Imaging Laboratory, Department of Electrical Engineering, California Institute of Technology, 1200 East California Boulevard, Pasadena, CA 91125 USA

## Abstract

We have developed a single-breath-hold photoacoustic computed tomography (SBH-PACT) system to reveal detailed angiographic structures in human breasts. SBH-PACT features a deep penetration depth (4 cm in vivo) with high spatial and temporal resolutions (255 µm in-plane resolution and a 10 Hz 2D frame rate). By scanning the entire breast within a single breath hold (~15 s), a volumetric image can be acquired and subsequently reconstructed utilizing 3D back-projection with negligible breathing-induced motion artifacts. SBH-PACT clearly reveals tumors by observing higher blood vessel densities associated with tumors at high spatial resolution, showing early promise for high sensitivity in radiographically dense breasts. In addition to blood vessel imaging, the high imaging speed enables dynamic studies, such as photoacoustic elastography, which identifies tumors by showing less compliance. We imaged breast cancer patients with breast sizes ranging from B cup to DD cup, and skin pigmentations ranging from light to dark. SBH-PACT identified all the tumors without resorting to ionizing radiation or exogenous contrast, posing no health risks.

## Abstract (web summary)

The current standard for breast cancer diagnostic is a mammogram; however, the sensitivity of mammography can be low in radiographically dense breasts. Here the authors develop a single-breath-hold photoacoustic computed tomography (SBH-PACT) system to reveal detailed angiographic structures in human breasts allowing the detection of higher blood vessel densities associated with tumors.

## Introduction

Breast cancer is the second most common cancer to affect women in the U.S. and is the second ranked cause of cancer-related deaths. About 1 in 8 (12%) women in the U.S. will develop invasive breast cancer during their lifetime^1^. Multiple large prospective clinical trials have demonstrated the importance of early detection in improving breast cancer survival^2–4^. While mammography is currently the gold standard used for breast cancer screening, it utilizes ionizing radiation and has lower sensitivity in women with dense breasts^5, 6^. Ultrasonography has been used as an adjunct to mammography, but suffers from speckle artifacts and low specificity^7, 8^. Magnetic resonance imaging (MRI) poses a large financial burden and requires the use of intravenous contrast agents that can cause allergy^9^, kidney damage^10^, and permanent deposition in the central nervous system^11^. Diffuse optical tomography has been investigated to provide functional optical contrast. However, the spatial resolution of the current prototypes limits their clinical use^12, 13^. Overall, each modality has notable advantages and limitations. Photoacoustic computed tomography (PACT) is a promising complementary modality that overcomes many of these limitations.

PACT—ultrasonically imaging optical contrast via the photoacoustic effect—breaks through the ~1 mm optical diffusion limit on penetration for high-resolution optical imaging in deep tissues^14, 15^. It combines the functional optical contrast of diffuse optical tomography and the high spatial resolution of ultrasonography. The rich contrast bestowed by optical absorption, which is related to various intrinsic and extrinsic contrast origins, enables PACT to perform structural, functional, and molecular imaging^16^. When a short-pulsed laser irradiates biological tissues, wideband ultrasonic waves (referred to as photoacoustic waves) are induced by transient thermoelastic expansion. The photoacoustic (PA) waves are then simultaneously measured by ultrasonic transducers around the tissue and are used to reconstruct the optical absorption distribution in the tissue^17^. In the near-infrared (NIR) region, the 1/e attenuation coefficient (1.0–1.3 cm^−1^)^18^ for light in an average breast is less than twice that for mammographic X-rays (0.5–0.8 cm^−1^)^19^. However, the optical absorption contrast of soft tissue is much higher than X-ray contrast^20^. For breast imaging, PACT can exploit these advantages to the fullest, offering high spatial and temporal resolutions with sufficiently deep nonionizing optical penetration^21, 22^. As the principal optical absorber in the NIR region, hemoglobin provides an endogenous contrast for imaging of blood vessels. A high density of blood vessels should correlate with angiogenesis^23–25^, which plays an important role in tumor growth and metastasis^26^.

Several breast PACT systems have been developed, employing different light illumination and detection schemes^27–36^. These systems have advanced PACT toward clinical application, but ongoing limitations remain to be addressed. Here, we consider five main factors: (1) sufficient penetration depth to accommodate most breast sizes and skin colors, (2) high spatial resolution to reveal detailed angiographic structures, (3) high temporal resolution to minimize motion artifacts and enable dynamic or functional studies, (4) minimal limited-view artifacts, and (5) sufficient noise-equivalent sensitivity and contrast-to-noise ratio to detect breast masses.

Specifically, the current systems’ limitations mainly arise from their long scanning times^27–29^ and/or limited-view apertures (i.e., missing data or a <2π steradian solid angle)^29–35^. Toi et al. recently reported a photoacoustic imaging system with a hemispherical detector array^27^, which was modified from a previous design^28^. Although the design for acoustic detection is slightly different, both used a sparse hemispherical detector array and scanned in a spiral pattern on a plane. The dense sampling, and the nearly isotropic 3D spatial resolutions produced elegant vascular images, but tumor detection was limited by respiratory motion artifacts resulting from the long scanning time (~4 min). Although co-registration partially mitigated the breathing motion distortion, the non-rigidity of the breast compromised the effectiveness. While larger vessels were coregistered, small tumor vessels, which often occur in small clusters, could be challenging to be imaged with partial data and even more difficult to be coregistered. Other groups have used planar transducer arrays^30–33^ and arc-shaped arrays^34^ for breast imaging. However, the limited views of these systems decreased their overall performances^37, 38^. Consequently, most blood vessels were not well visualized in their images. The same problem occurred with linear transducer arrays, either fixed in position^35^ or scanned^29^. A ring-shaped array of 32 elements was developed at presumably relatively low system cost^36^. However, the low number of elements severely limited the field of view due to the spatial Nyquist sampling criterion, resulting in degradation of image quality^39^.

Here, we report a significant advancement in breast PACT technology that overcomes all of the aforementioned limitations. Our breast imaging modality—single-breath-hold PACT (SBH-PACT)—is the first PACT system that meets the aforementioned five conditions: (1, 2) Combining 1064-nm light illumination and a 2.25-MHz unfocused ultrasonic transducer array, SBH-PACT achieved up to 4 cm in vivo imaging depth and a 255 µm in-plane resolution (approximately four times finer than that of contrast-enhanced MRI^40^). (3) Equipped with one-to-one mapped signal amplification and data acquisition (DAQ) circuits, SBH-PACT can obtain an entire 2D cross-sectional breast image with a single laser pulse, or obtain a volumetric 3D image of the entire breast by fast elevational scanning within a single breath-hold (~15 s). The 10 Hz 2D frame rate, currently limited by the laser repetition rate, enables SBH-PACT to observe biological dynamics in a cross-section associated with respiration and heartbeats without motion artifacts. (4) A full-ring 512-element ultrasonic transducer array enables SBH-PACT for full-view fidelity in 2D imaging planes and delivers high image quality. (5) Capitalizing on the optimized illumination method and signal amplification, SBH-PACT achieves sufficient noise-equivalent sensitivity to clearly reveal detailed angiographic structures both inside and outside breast tumors without the use of exogenous contrast agents.

In this pilot study, SBH-PACT was used to image one healthy volunteer and seven breast cancer patients. SBH-PACT clearly identified eight of the nine breast tumors by delineation of angiographic anatomy. These tumors were subsequently verified by ultrasound-guided biopsy. In addition, to improve on the interpretation of images, we developed an algorithm to highlight tumors automatically. Tumors were clearly revealed by SBH-PACT in all breasts even in radiographically dense breasts, which could not be readily imaged by mammography. Taking advantage of the high imaging speed, we demonstrated elastographic SBH-PACT for tumor detection by assessing deformations caused by breathing. SBH-PACT elastography identified the tumor missed in angiographic imaging, and thus improved the sensitivity of tumor detection. At such high spatiotemporal resolutions, SBH-PACT is able to differentiate arteries from veins by detecting blood flow-mediated arterial deformation at the heartbeat frequency.

## Results

### SBH-PACT of healthy breast anatomy and dynamics

**Fig. 1:**
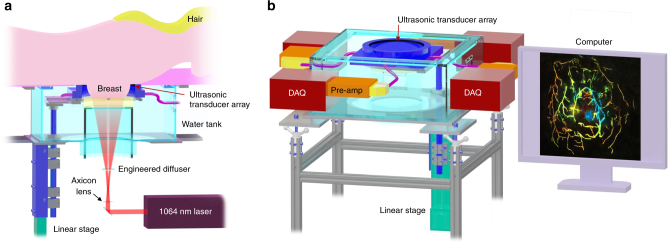
Representations of the SBH-PACT system. **a** Perspective cut-away view of the system with data acquisition components removed. **b** Perspective view of the system with patient bed and optical components removed. DAQ data acquisition system, Pre-amp pre-amplifier circuits

The SBH-PACT system is placed underneath a patient bed with minimal separation from the top surface of the bed to the top scanning position of the ultrasonic transducer array (Fig. 1a and Supplementary Fig. 1). With the patient lying prone on the bed, the breast to be imaged is slightly compressed against the chest wall by a soft agar pillow. Compared to craniocaudal or mediolateral breast compression, compression against the chest wall not only avoids pain, but also gives the least thickness breast tissue for light to penetrate from the nipple to the chest wall. The laser illuminates the breast from beneath the patient’s breast, and the ultrasonic transducer array detects photoacoustic waves circumferentially around the breast. The light beam is converted into a donut shape via an axicon lens followed by an engineered diffuser. Compared to a Gaussian beam, the donut beam provides more uniform illumination inside the breast (Supplementary Fig. 2) and also deposits less energy on the nipple and areola, which have a higher concentration of pigment. We take advantage of the low optical attenuation of 1064 nm light to achieve sufficient optical penetration in breast tissue^41^.

To provide 2D panoramic in-plane acoustic detection, we employ a full-ring ultrasonic transducer array with 512 elements. Four sets of 128-channel data acquisition systems provide simultaneous one-to-one mapped associations with the 512-element transducer array (Fig. 1b and Supplementary Fig. 1). Therefore, we acquire photoacoustic signals from a cross section within 100 µs without multiplexing after each laser pulse excitation (Methods). The ultrasonic transducer elements have a central frequency of 2.25 MHz and a one-way bandwidth of more than 95% (Supplementary Fig. 3), providing an experimentally quantified in-plane resolution of 255 µm (Supplementary Fig. 4). The height of each transducer element yields a moderate divergence angle in the elevational direction (~9.0˚ full width at half maximum (FWHM)), yielding a flared diffraction pattern (Supplementary Fig. 5). This pattern enables both 2D imaging of a breast cross section per laser pulse and 3D imaging of the whole breast by scanning elevationally (Methods). Our 3D back-projection algorithm can reconstruct a volumetric image with an elevational resolution of 5.6 mm, which is ~3 times finer than that given by the 2D reconstruction algorithm (Supplementary Fig. 5 and Supplementary Movie 1).

**Fig. 2:**
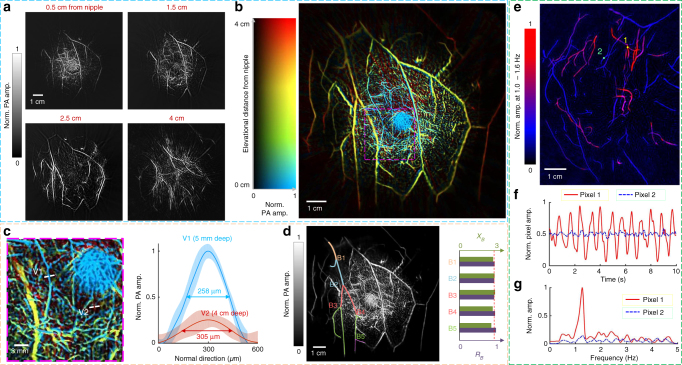
SBH-PACT of healthy breasts. **a** Vasculature in the right breast of a 27-year-old healthy female volunteer. Images at four depths are shown in increasing depth order from the nipple to the chest wall (also see Supplementary Movie 2). **b** The same breast image with color-encoded depths. **c** A close-up view of the region outlined by the magenta dashed box in **b**, with selected thin vessels and their line spread plots. **d** A selected vessel tree with five vessel bifurcations, labeled from B1 to B5. At each bifurcation, the diameter relationships between the parent vessel (*D*_parent_) and daughter vessels (*D*_daughter_) are presented on the right. *X*_*B*_ is the junction exponent, and *R*_*B*_ is defined as $R_B = D_{{\rm parent}}^3/(D_{{\rm daughter}\_{\rm a}}^3 + D_{{\rm daughter}\_{\rm b}}^3)$. **e** Heartbeat-encoded arterial network mapping of a breast cross-sectional image (red = artery, blue = vein). **f** Amplitude fluctuation in the time domain of the two pixels highlighted by yellow and green dots in **e**. The pixel value in the artery shows changes associated with arterial pulse propagation (also see Supplementary Movie 3). **g** Fourier domain of the pixel value fluctuations in **f**. The oscillation of the arterial pixel value shows the heartbeat frequency at ~1.2 Hz

Before imaging breast cancer patients, the performance of SBH-PACT was assessed by imaging a 27-year-old healthy female volunteer. By scanning the transducer array elevationally through her right breast, within one breath hold (~15 s), we revealed the angiographic anatomy from the nipple to the chest wall (Fig. 2a and Supplementary Movie 2). The color-encoded depth-resolved image clearly revealed the detailed angiographic structures of the entire breast (Fig. 2b), visualizing the vasculature down to an apparent vascular diameter of 258 µm (Fig. 2c). To accurately measure the vascular diameters, we identified vessel boundaries in different slices through a correlation-based template matching method^42^ (Supplementary Fig. 6 and Methods). By doing this, we further investigated the relationship between parent and daughter vessels at vascular bifurcations, which is expressed by the junction exponent (*X*_*B*_)^43^. We selected a vessel tree in the breast and marked five branch levels with distinct colors (Fig. 2d). At five vascular bifurcations (B1–B5), we calculated the junction exponents as well as the ratios between the cube of the diameter of the parent vessel and the sum of the cubes of the diameters of the daughter vessels (Fig. 2d). For the eight subjects (one healthy volunteer and seven breast cancer patients), we picked five vascular bifurcations in each of their breasts and quantified the average junction exponent (Supplementary Fig. 7), which has a mean value of 2.63 ± 0.34. The junction exponents generally decrease with increasing age^44, 45^.

During a breath hold within 10 s, we imaged a cross section of the contralateral healthy breast in one of the breast cancer patients. Working in 2D mode at 10 Hz frame rate, SBH-PACT continuously monitored arterial pulsatile deformation inside the breast (Supplementary Movie 3) by fixing the transducer array at a specific elevational position^46^. PA signals were analyzed pixel-wise in the frequency domain to identify arteries and veins according to the heartbeat frequency (see Methods) (Fig. 2e). For illustration, we selected a pixel from one artery and one vein (highlighted by round dots 1 and 2 respectively in Fig. 2e) and plotted their pixel value fluctuation (Fig. 2f). The periodic oscillation of the pixel values in the artery indicates that the changes were the result of pulse waves propagating through the arterial network. The oscillation frequency further reveals the subject’s heart rate of ~1.2 Hz (Fig. 2g). Considering that arterial blood has a relatively narrow range of oxygen saturation (sO_2_)^47^, average PA signals from arteries can potentially be used to calibrate the local optical fluence (mJ cm^−2^) deep in the breast, and thus enable accurate quantification of functional parameters (e.g., blood sO_2_) with an additional laser wavelength (e.g., 750 nm)^48, 49^.

### SBH-PACT of breast cancer anatomy, segmentation, and elastography

**Fig. 3:**
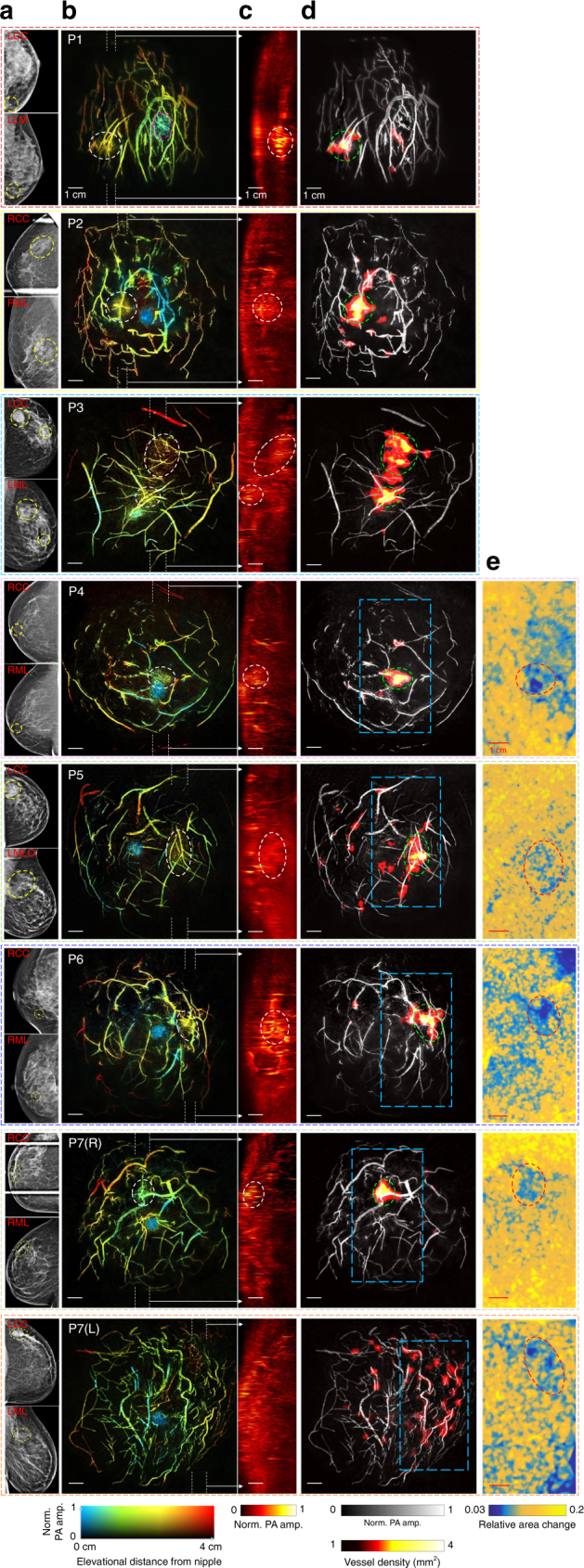
SBH-PACT of cancerous breasts. **a** X-ray mammograms of the affected breasts of seven breast cancer patients. LCC left cranial-caudal, LLM left lateral-medio, LML left mediolateral, LMLO left mediolateral-oblique, RCC right cranial-caudal, RML right medio-lateral. **b** Depth-encoded angiograms of the eight affected breasts acquired by SBH-PACT. Breast tumors are identified by white circles. For illustration, we marked the nipple of the first patient (P1) with a magenta circle. P1—48-year-old female patient with an invasive lobular carcinoma (grade 1/3); P2—70-year-old female patient with a ductal carcinoma in situ (microinvasion grade 3/3); P3—35-year-old female patient with two invasive ductal carcinomas (grade 3/3); P4—71-year-old female patient with an invasive ductal carcinoma (grade 3/3); P5—49-year-old woman with a stromal fibrosis or fibroadenoma; P6—69-year-old female patient with an invasive ductal carcinoma (grade 2/3); P7—44-year-old female patient with a fibroadenoma in the right breast and an invasive ductal carcinoma (grade 2/3) in the left breast. **c** Maximum amplitude projection (MAP) images of thick slices in sagittal planes marked by white dashed lines in **b**. **d** Automatic tumor detection on vessel density maps. Tumors are identified by green circles. Background images in gray scale are the MAP of vessels deeper than the nipple. **e** Maps of the relative area change during breathing in the regions outlined by blue dashed boxes in the angiographic images in **d**. The same tumors are identified by red circles. The elastographic study began with Patient 4, and it revealed all imaged tumors, including the undetected one in **d** (P7(L))

The improved noise-equivalent sensitivity (Supplementary Fig. 8) enabled SBH-PACT to detect breast tumors with fine details, making this imaging modality potentially useful for multiple applications in breast clinical care. We imaged seven breast cancer patients (Fig. 3), with breast sizes ranging from B cup to DD cup (over 99% of the U.S. population has breast sizes of DD cup or smaller^50^) and skin pigmentations ranging from light to dark (Methods). Angiogenesis, which plays a central role in breast cancer development, invasion, and metastasis, is the essential hallmark by which SBH-PACT differentiates lesions from normal breast tissue^23–25^. Well correlated with the tumor locations shown in mammograms and reported by ultrasound-guided biopsy (Fig. 3a and Methods), SBH-PACT showed eight of the nine tumors by observing higher blood vessel densities associated with tumors in the depth-encoded images (Fig. 3b and Supplementary Fig. 9). We further selected tumor-containing slices perpendicular to the chest wall (marked by white dashed lines in Fig. 3b). In these sagittal (side-view) images, the same tumors, where higher PA amplitude is shown, can be seen at corresponding locations (Fig. 3c). In the X-ray mammograms of Patient 1 (P1) and Patient 6 (P6), the lesions in the dense breasts are barely distinguishable. In comparison, SBH-PACT clearly revealed the tumors not readily seen in mammograms, not withstanding the high radiographical density of the breast.

To assist in translation of the technology to a clinical setting, we developed a tumor segmentation algorithm to distinguish tumors automatically. Presumably due to angiogenesis, tumors appear as regions of denser blood vessels in SBH-PACT images (Fig. 3b). To segment tumors automatically, we extracted the vessel skeleton and produced a vessel density map of the breast (local vessel number / local area). The regions with the highest vessel density highlight the breast tumors (Fig. 3d and Methods).

In addition to direct observation of blood vessel density, SBH-PACT detected the difference in compliance between tumors and surrounding normal breast tissue, providing an alternate concurrent contrast to detect breast cancer. Before performing elastography on breast cancer patients, we demonstrated this method on breast-mimicking phantoms (Methods and Supplementary Fig. 10). Working in 2D imaging mode, SBH-PACT quantified the relative area changes in a breast cross section when minor deformations were caused by breathing (Supplementary Movie 4). Because breast tumors are generally less compliant than normal breast tissue^51^, the regions with lower relative area changes indicated the breast tumor (Fig. 3e). Unlike ultrasonic elastography, SBH-PACT elastography utilized the contrast of hemoglobin and formed area-quantificational grids between vessels (Methods). From only angiographic anatomy detailed by SBH-PACT, the only tumor we missed was located in a marginal region of a D cup breast (P7(L)), where light illumination was insufficient. However, with the addition of SBH-PACT elastography, the missed tumor was identified. Taking advantage of the short time requirement for elastographic measurement (~10 s), SBH-PACT can observe both blood vessel density and tissue compliance simultaneously within ~30 s. Taken together, these two measurements can improve the sensitivity of breast cancer detection.

### Statistics

In this pilot study, SBH-PACT identified eight of the nine biopsy-verified tumors by assessing blood vessel density. Moreover, the initially undetected tumor was subsequently revealed by elastographic SBH-PACT. Pathology reports showed two benign tumors (Patient 5, stromal fibrosis or fibroadenoma; Patient 7, right, fibroadenoma), one ductal carcinoma in situ (DCIS) with a 3/3 nuclear grade (Patient 2), and six invasive carcinomas (all other cases).

**Fig. 4:**
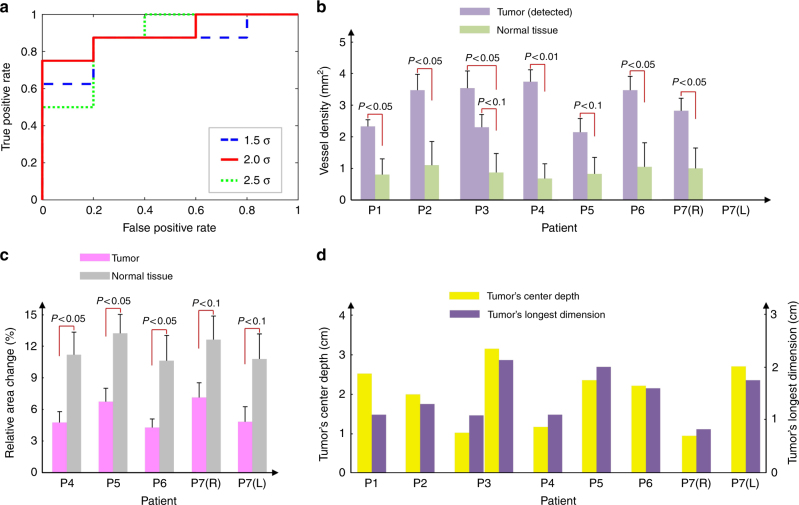
Statistics. **a** The ROC curves of breast tumor detection based on blood vessel density. *σ*, standard deviation. **b** The average vessel density in each tumor and surrounding normal breast tissue. **c** The relative area change in each tumor and surrounding normal breast tissue caused by breathing. The elastographic study was started with Patient 4. **d** The longest dimension and center depth of each tumor

Angiogenesis serves as a basis for tumor identification. Considering the diversity among the subjects, we defined high blood vessel densities as values greater than the whole-breast average plus (a) 1.5, (b) 2.0, or (c) 2.5 times the standard deviation, respectively. We calculated and compared the ratios of average vessel density between the high-density region and the normal-density region in each affected and contralateral breast (Supplementary Fig. 11). Receiver operating characteristic (ROC) curves (Fig. 4a) were plotted by varying the threshold of the ratios from 1 to 6. Based on the data from the finite set of subjects, option (b) yielded the largest area (0.90) under the ROC curve. A threshold within (2.26, 2.58) produced a sensitivity (true positive rate) of 88% and a specificity (true negative rate) of 80%. We further performed training and testing studies by obtaining a threshold based on randomly picked six breasts (training set) and then applying the threshold to the remaining seven breasts (testing set). We repeated this procedure ten times and calculated the average sensitivity and specificity (Supplementary Table 1).

We then demarcated tumors in each breast and computed the average vessel densities inside and outside the tumors (Methods). The average vessel density ratios between the tumors and the surrounding normal breast tissues were 3.4 ± 0.99 (Fig. 4b). In addition, the mean of the average vessel density ratios of the six malignant tumors was 1.4 times higher than that of the two benign ones (Supplementary Fig. 12).

Since the elastography study began with Patient 4, SBH-PACT elastography identified all five tumors in the subsequent four patients (Fig. 4c). The average breath-induced area change in tumors was around 2 times lower than that in normal breast tissue. As the patient recruitment protocol excluded patients with a mass smaller than 1 cm in diameter in this pilot study, the longest dimension of the smallest tumor we detected was approximately 0.8 cm (Fig. 4d). This tumor was located in the right breast of Patient 7, who was recruited due to a larger tumor in her left breast. However, with 255 µm spatial resolution and refined noise-equivalent sensitivity, SBH-PACT has the potential to detect smaller breast cancers once angiogenesis sufficiently progressed. Patient 3 had DD cup breasts, and her breast was compressed against the chest wall to roughly a cylinder. The tumor in her breast had a depth of ~3.2 cm (elevational distance from the nipple), which was the deepest among the recruited patients (Fig. 4d).

## Discussion

We have developed an SBH-PACT system that integrates deep penetration, high spatiotemporal resolution, sensitive breast cancer detection, and 2D/3D switchable modes. One-to-one mapped low-noise amplifiers and DAQ circuits enabled 2D imaging using a single laser impulse or 3D imaging of an entire breast within a single breath hold (~15 s). The high imaging speed avoided respiration-induced motion artifacts and enabled detection of breast tumors by detailing tumor associated angiogenesis. The donut-shaped optical illumination and panoramic acoustic detection provided a more uniform fluence distribution in deep tissue and best in-plane coverage of ultrasound reception, respectively, delivering high image quality. Furthermore, considering the low cancer detection rate (0.41%)^52^, even though modern mammography uses a low dose of ionizing radiation, the risk-to-benefit ratio (e.g., 8–17% https://www.ncbi.nlm.nih.gov/pubmed/11388061 for 40–50 year-old women)^53, 54^ is considered high. In comparison, SBH-PACT requires neither ionizing radiation nor an exogenous contrast agent, yielding zero risk.

In SBH-PACT, the laser beam was broadened into a donut shape with an outer diameter of ~10 cm, depositing light with an average laser fluence of ~20 mJ cm^−2^ on the breast surface (only 1/5 of the American National Standards Institutes safety limit)^55^. This outer radius covered most breasts and provided satisfactory SNR in breast images. Merely assessing blood vessel density, we missed one tumor, which was located in an insufficiently illuminated marginal region of a D cup breast (P7(L) in Fig. 3a). Therefore, SBH-PACT can potentially improve sensitivity further in breast cancer detection if equipped with a more energetic laser, which will allow us to enlarge the illumination area and increase the optical fluence.

Our automatic tumor segmentation algorithm made it easier to recognize tumors by highlighting the suspicious affected region with the highest vessel density. In addition, the high 2D imaging speed of SBH-PACT (10 Hz frame rate) enabled us to perform elastographic measurements and to improve on breast cancer detection. The capability of SBH-PACT to map arterial distribution can potentially be useful in diagnosing artery-related diseases^56–58^. In addition, the knowledge of vessel diameters and average PA signals from arteries can be used to calibrate the local optical fluence^48, 49^, thus providing accurate spectral sO_2_ measurement in deep tissue.

By developing this advanced breast imaging modality, we provided a promising tool for future clinical use including not only screening, but also diagnostic studies to determine extent of disease, to assist in surgical treatment planning, and to assess responses to neoadjuvant chemotherapy. Compared to mammography, SBH-PACT utilizes non-ionizing radiation, shows early promise for sensitivity in radiographically dense breasts, and imposes less or no pain by only slightly compressing the breast against the chest wall. Because the average hemoglobin concentration in malignant tumors is generally twice that in benign tumors^59–61^, SBH-PACT has the potential to distinguish malignant tumors from benign tumors by quantifying blood vessel densities in the tumor (Supplementary Fig. 12). Based on the vessel density in the two benign tumors and the six detected malignant ones in this study, the threshold of the vessel density ratio between tumors (either malignant or benign) and healthy tissues should be set within the range of (2.72, 2.76) to optimally differentiate malignant tumors from benign ones; however, more patients need to be imaged to achieve statistical significance. Using hemoglobin as the contrast, SBH-PACT can potentially monitor breast cancer’s response to neoadjuvant chemotherapy by acquiring information similar to that of contrast-enhanced MRI, yet with finer spatial resolution, higher imaging speed, and only endogenous contrast.

## Methods

### System construction

The SBH-PACT system mainly comprises of an illumination laser, an ultrasonic transducer array, signal amplification/acquisition modules, a linear scanning stage, and a patient bed (Fig. 1). The 1064-nm laser beam (PRO-350-10, Quanta-Ray, 10-Hz pulse repetition rate, 8–12-ns pulse width) was first passed through a lab-polished axicon lens (25 mm diameter, 160° apex angle), then expanded by an engineered diffuser (EDC-10-A-2 s, RPC Photonics) to form a donut-shaped light beam. The laser fluence (20 mJ cm^−2^) was within the American National Standards Institutes (ANSI) safety limit for laser exposure (100 mJ cm^−2^ at 1064 nm at a 10-Hz pulse repetition rate)^55^. To synchronize the SBH-PACT system, the laser’s external trigger was used to trigger both the data acquisition systems and the linear scanner.

To achieve 2D panoramic acoustic detection, we employed a 512-element full-ring ultrasonic transducer array (Imasonic, Inc.; 220 mm ring diameter; 2.25 MHz central frequency; more than 95% one-way bandwidth; Supplementary Fig. 3). Each element had a flat-rectangular aperture (5 mm element elevation size; 1.35 mm pitch; 0.7 mm inter-element spacing). The ultrasonic transducer array housing was mounted on a stainless steel rod (25 mm diameter) and enclosed in an acrylic water tank. A linear stage (THK America, Inc., KR4610D) was fixed beneath the water tank and moved the transducer array elevationally via the stainless steel rod. Four sets of lab-made 128-channel preamplifiers (26 dB gain) were placed around the water tank, connected to the ultrasonic array housing via signal cable bundles. Each set of preamplifiers was further connected to a 128-channel data acquisition system (SonixDAQ, Ultrasonix Medical ULC; 40 MHz sampling rate; 12 bit dynamic range) with programmable amplification up to 51 dB. The digitized radio frequency data were first stored in an onboard buffer, and then transferred to a computer through a universal serial bus 2.0 (Supplementary Fig. 1). The data acquisition systems were set to record PA signals within 100 µs after each laser pulse excitation.

The patient is positioned prone with one breast dependent and placed into a large aperture in the bed. An agar pillow affixed on top of an acrylic tube lightly pressed the breast against the chest wall. The bed top was covered by cushioning memory foam. The water tank was fully filled with water preheated to a temperature of 35 °C. Both the patient bed and the SBH-PACT system were supported by T-slotted aluminum frames.

### Half-time PA reconstruction in 2D and 3D modes

We used the half-time universal back-projection (UBP) algorithm^62^ to reconstruct all images in this work. In 2D imaging mode, the time-domain PA signals generated by each laser pulse were back-projected to a 2D imaging plane. Determined by the acoustic divergence angle (~9.0˚) at FWHM in the elevational direction (Supplementary Fig. 5), the elevational resolution at the center was ~16.1 mm.

Alternatively, when working in 3D mode, the ultrasonic transducer array scanned the entire breast from the chest wall to the nipple. The time-domain PA signals acquired at all elevational scanning steps were then back-projected simultaneously into the 3D space. To accommodate the acoustic divergence angle in the elevational direction, 3D-UBP added a weight to the back-projected PA signals at different elevational divergence angles (Supplementary Fig. 5). To accurately reconstruct objects in the Fraunhofer zone, we back-projected PA signals from virtual transducers located at the transition points between the Fresnel and Fraunhofer zones^63^. Sharing the same in-plane resolution as the 2D mode, 3D-UBP provided an improved elevational resolution of 5.6 mm.

The full-ring transducer array with 512 elements could spatially well sample objects — according to the spatial Nyquist criterion — within a field of view (FOV) of ~39 mm in diameter^39^. To eliminate aliasing caused by under-sampling in regions outside of this FOV, we low-pass filtered PA signals with cut-off frequencies determined by the distance to the center of the ring array.

Each volumetric image was first reconstructed with a voxel size of 1 mm in the elevational direction and 0.1 × 0.1 mm^2^ on the horizontal plane. We then batch-processed all the reconstructed images to improve contrast (batch processing before vesselness filtering in Supplementary Fig. 9). In each horizontal slice, we applied Hessian-based Frangi vesselness filtration^39^ to enhance the contrast of blood vessels with diameters ranging from 3 to 12 pixels. In each filtered slice, adaptive thresholding was used to segment blood vessels^42^, followed by morphology filtration for single-pixel elimination. In the elevational direction of each filtered volumetric image, we selected voxels with the largest PA amplitudes and then projected their depths to form a 2D image. We applied median filtration with a window size of 3 × 3 pixels to the depth image. Another median filtration with a window size of 6 × 6 pixels was further applied inside the segmented vessels to the segmented vessels’ depths. Different RGB (red, green, blue) color values were assigned to discrete depths (vertical color bar in Fig. 2b). Finally, the 2D depth-resolved color-encoded image was multiplied by the MAP image pixel by pixel to represent the maximum amplitudes (horizontal color bar in Fig. 2b). To further reduce noise and improve image quality, we also tuned the above parameters in 2D slices at different depths (custom processing in Supplementary Fig. 9). As shown in Supplementary Fig. 9, the structures in all three sets of images match well with each other, showing the fidelity of the vesselness filtering and custom processing.

### Vascular diameter measurement

Vascular diameters were accurately measured by identifying vessel boundaries through a correlation-based template matching method^42^. The templates were generated through simulation (Supplementary Fig. 6). The impulse responses of all ultrasonic transducers were used to simulate the images of vessels with different sizes (0.5–2.0 mm) and orientations. The diameters of vessels chosen from the SBH-PACT breast images were quantified by matching the reconstructed vessel images with the generated templates.

### Arterial vessel mapping

Working in 2D imaging mode, SBH-PACT was able to monitor blood flow-mediated arterial fluctuation (Supplementary Movie 3). After removing displacement through rigid transformation, we analyzed the pixel value fluctuation during a patient’s breath hold (~10 s). We found that arteries fluctuated much more than veins at the frequency of the heartbeat. The fluctuation of the pixel values in the artery indicated the changes associated with arterial pulse propagation (Fig. 2g).

To separate fluctuations caused only by heart beats, frames with strong motion caused by body movement were first removed. The entire imaging field was then divided into 16 slightly overlapping subdomains. In each subdomain, we chose the first frame as the reference frame; other frames were registered to it through rigid transformation, optimizing the frame–frame correlation. In each subdomain, a Gaussian filter with a radius of 0.2 mm was applied to all registered frames to reduce high spatial-frequency noise. We then applied Fourier transformation to each pixel’s value through all the frames. The fluctuations in pixel values induced by arterial pulse propagation were quantified within the frequency range (1.0–1.6 Hz) of heartbeat cycles^64^.

### Tumor segmentation

SBH-PACT showed breast masses by revealing a greater density of blood vessels, presumably due to angiogenesis, in tumor regions. To segment tumors automatically, we extracted the vessel skeleton and produced a vessel density (number of vessels / area) map of the breast. The regions with the highest vessel density highlighted the breast mass of interest (Fig. 3d).

The dense vessels in the nipple would affect the automatic tumor segmentation. Therefore, the shallowest slices containing the nipple were first removed. The remaining slices were used to generate the MAP image. A vessel mask was generated from the MAP by Hessian filtering and threshold-based segmentation. Based on the mask, vessel centerlines were extracted by removing boundary pixels. The vessel centerlines were broken into independent vessels at junction points. To reduce noise further, we removed independent vessels with lengths less than 3 pixels (255 µm spatial resolution divided by 100 µm pixel size is approximately 3). A 2 mm × 2 mm window was then used to scan the entire image. At each scanning location, the number of vessels (independent segments) inside the window was counted and assigned to the center pixel in the window. The vessel density was quantified as the number of vessels divided by the window area. To compute the average vessel density of the whole breast, we included pixels inside a 10 cm-diameter circle around the image center.

To demarcate breast tumors from MAP images, we first identified suspicious regions where blood vessel densities were higher than a threshold, which was set to each whole-breast’s average plus 2.0 times the standard deviation. Among the eight affected breasts, the smallest suspicious region had a diameter of 1 mm. We then counted the numbers of pixels in each contiguous region and rejected the regions with pixel counts fewer than 1855 (18.55 mm^2^) to eliminate false positive cases (Supplementary Fig. 13). The remaining contiguous regions were labeled as tumors and the smallest one had a longest dimension of 8 mm (Fig. 4d). In comparison, contrast-enhanced MRI on a 1.5 Tesla scanner can detect breast tumors as small as 4 mm, which is similar to the smallest size of tumors detectable by X-ray mammography^65, 66^.

### Elastographic study

SBH-PACT’s high imaging speed enabled differentiation in compliance between tumors and surrounding normal breast tissues, providing another contrast for detecting breast cancer. We first performed SBH-PACT elastographic measurements on a breast phantom. The phantom comprised a ball with 7% agar (mimicking breast tumor) embedded in a base of 2% agar (mimicking normal breast tissue)^67^. Chopped human hair was uniformly distributed in the phantom to mimic small blood vessels. Working in 2D imaging mode, SBH-PACT quantified the relative area changes in a cross section when minor deformations were induced by periodic compressions (~0.25 Hz) on top of the phantom. Due to the low elevational sectioning power of 2D imaging, objects in 2D frames were mainly influenced by coronal dilation instead of elevational displacement. Accordingly, SBH-PACT elastography clearly revealed the agar ball with correct size and location (Supplementary Fig. 10). No obvious differences were observed in the concentration of the hair fiber between the balls and the phantom base.

To assess deformations over time, the first frame was taken as a reference. Other frames were registered to the first frame through a non-rigid demon algorithm^68^ in Matlab. For each pixel of registered frames, the standard deviation (STD) of the value variations was calculated. Pixels with relatively small STDs were stably registered and were used for deformation quantification. The entire image was then segmented into 2 mm × 2 mm squares. One stably registered pixel was chosen from each square, and triangular grids were further generated from these registered pixels. The triangular grids were mapped back to the original unregistered frames, and their areas were calculated. For each grid, Fourier transformation was applied to quantify the area variation at the frequency of periodic compression, and amplitudes were assigned to the pixels inside this triangle to generate the deformation map. To further reduce noise, 100 deformation maps were generated with randomly registered pixels in the squares. The final image is the average of the 100 deformation maps.

To conduct SHB-PACT elastography of the breast, patients were asked to breathe normally. The chest wall pushed the breast against the agar pillow, elevationally generating a deformation of the breast in the coronal plane. We used the same method to quantify the change of area between blood vessels in the breast. Tumors, being stiffer, could be identified in areas with less deformation than normal breast tissue.

### Patient recruitment and human experiment protocols

All the human experiments followed protocols approved by the Institutional Review Board (IRB) and Protocol Review and Monitoring Committee (PRMC) of Washington University in St. Louis. We enrolled one healthy volunteer and seven female patients with consent documents signed. All recruited patients met the following inclusion criteria: (1) Patients were newly diagnosed with breast tumors highly suspicious for malignancy, larger than 1 cm in diameter, and were eligible for percutaneous biopsy; (2) Patients were females >18 years of age; (3) Patients were able to understand and willing to sign a written informed consent document. The exclusion criteria included (1) patients whose weight exceeded 300 lbs (the weight limit of the steps for mounting the bed); (2) patients who were pregnant; (3) patients with uncontrolled intercurrent illness including, but not limited to, ongoing or active infection of the breast and/or axilla, symptomatic congestive heart failure, unstable angina pectoris, cardiac arrhythmia, or psychiatric illness/social situations that would limit compliance with study requirements.

Photoacoustic imaging was performed after a standard of care (SOC) work-up, but in advance of percutaneous biopsy. This order of events was designed to minimize confounding imaging findings related to biopsy-induced hemorrhage. Patients underwent only one PACT imaging study, which took less than 10 min. We imaged both the contralateral and affected breasts. For the abnormal breast, we analyzed the tumor size, tumor depth, blood vessel density, and signal amplitude in the breast images. Our analysis of tumor size/depth was further compared with the standard imaging results (mammography and ultrasonography). To identify the tumor types and grades, histopathology results from the SOC biopsy were used as the ground truth for interpretation of the results.

### Standard of care work-up, percutaneous biopsy, and pathologic diagnosis

Patients were imaged at the Joanne Knight Breast Health Center at Siteman Cancer Center in partnership with Washington University School of Medicine (WUSM), Mallinckrodt Institute of Radiology. All standard of care imaging was performed by subspecialist breast imaging physicians who are faculty of WUSM. Using established clinical protocols, abnormalities were identified either through routine screening mammography, or diagnostic evaluation in symptomatic patients. Pre-biopsy work up included combinations of digital mammography, digital breast tomosynthesis, and ultrasound. Formal BI-RADS (breast imaging, reporting and data system) assessments were assigned in all cases, with appropriate recommendation for biopsy. Eligible patients were approached and informed consent obtained in advance of the biopsy. Image-guided percutaneous biopsy was obtained using real-time ultrasound guidance and a 12-guage or 14-guage spring-loaded biopsy needle (chosen at the discretion of the performing physician.). Core specimens were submitted in formalin to the pathology department for histologic analysis as per normal routine at the institution. All cases were reviewed following receipt of the final pathology report to determine radiologic-pathologic correlation. Some patients underwent contrast enhanced breast MRI following confirmation of malignancy.

### Data availability

All data are available within the Article and Supplementary Files, or available from the authors upon request.

## Electronic supplementary material

Supplementary Information

Description of Additional Supplementary Files

Supplementary Movie 1

Supplementary Movie 2

Supplementary Movie 3

Supplementary Movie 4
