# Supplementary material for: Single-breath-hold photoacoustic computed tomography of the breast
Source: Nat Commun. 2018 Jun 15;9:2352. doi: 10.1038/s41467-018-04576-z (PMC6003984; doi:10.1038/s41467-018-04576-z)
Supplement: Supplementary file 1 — Supplementary Information [file 41467_2018_4576_MOESM1_ESM.pdf]

Supplementary Information

Supplementary Figures

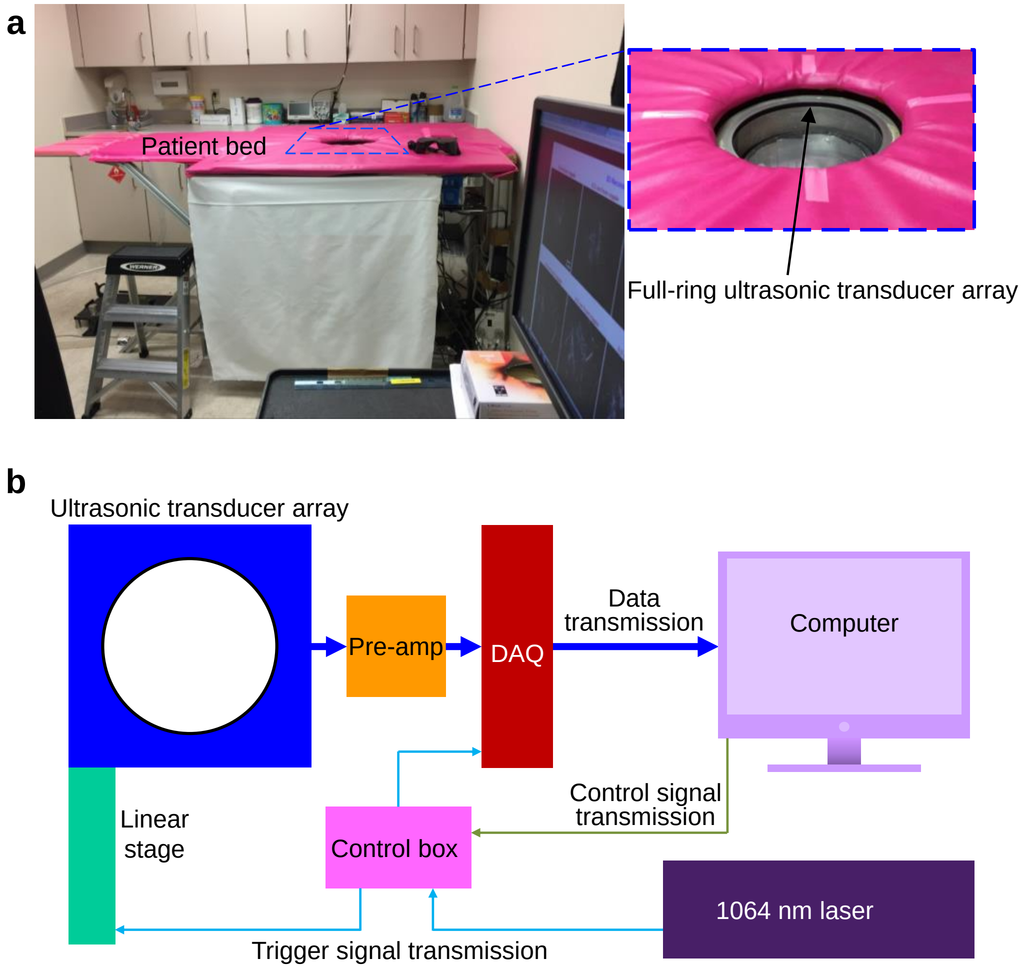

Supplementary Figure 1. Photograph and schematic of the SBH-PACT system. **a,** Photograph of the system, with a close-up view of the breast aperture in the bed. **b,** Signal flow diagram for the system.

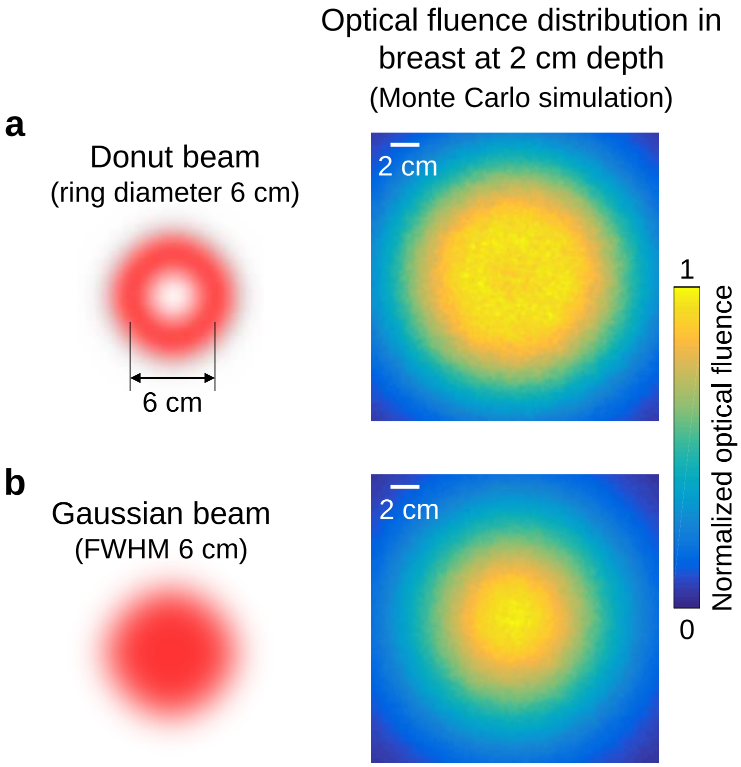

Supplementary Figure 2. Simulation of the optical fluence in breast tissue at 2 cm depth, produced by different illumination schemes. **a,** Distribution of the optical fluence in breast tissue when the illumination beam is donut-shaped. After removing the engineered diffuser, the ring diameter is approximately 6 cm. **b,** Distribution of the optical fluence in breast tissue at the same depth when the illumination beam is Gaussian-shaped. The FWHM of the beam is approximately 6 cm. To mimic a breast compressed against the chest wall, we built a cylindrical breast model with a height of 4 cm and a diameter of 15 cm. In this numerical model, the absorption coefficient (0.05 cm^{-1}) and the reduced scattering coefficient (7 cm^{-1}) inside the breast were selected for a 1064 nm wavelength[1].

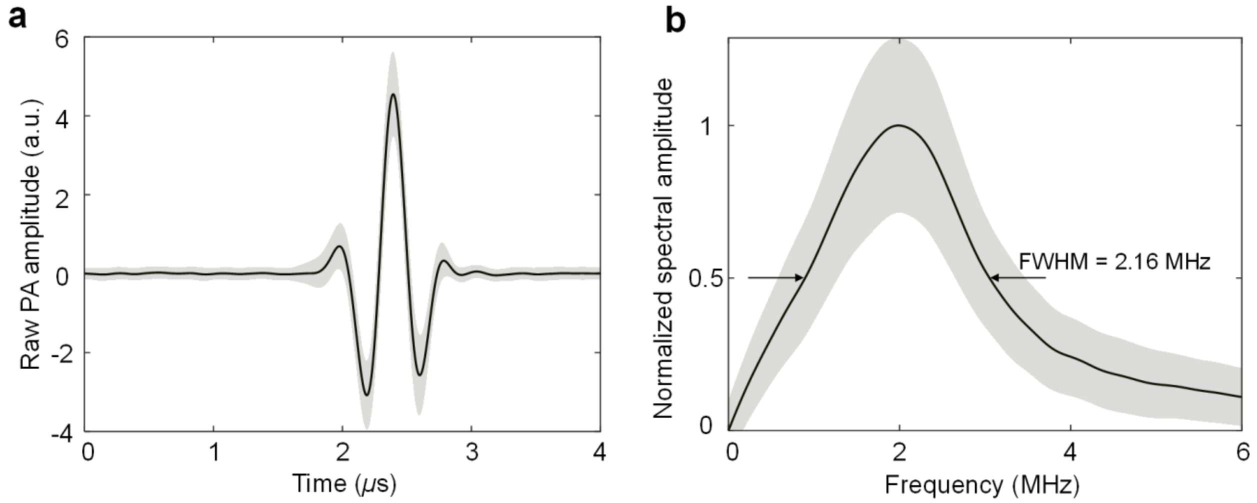

Supplementary Figure 3. Electrical impulse response (EIR) of the SBH-PACT system. **a,** The raw radio frequency (RF) signal from each ultrasonic transducer element corresponding to a point PA source at the center of the full-ring array. The black solid line represents the mean value of all transducer elements' responses, and the gray region represents the standard deviation across the elements. **b,** Fourier transform amplitude of each RF signal in **a**, showing that the bandwidth of the transducer array is about 2.16 MHz. The black solid line represents the mean value of the spectral amplitude of all RF signals, and the gray region represents the standard deviation across the elements. The point source was created by fixing a carbon particle (20–50 μm) in an agar phantom. The particle was small enough to be regarded as a spatial point source for the SBH-PACT system.

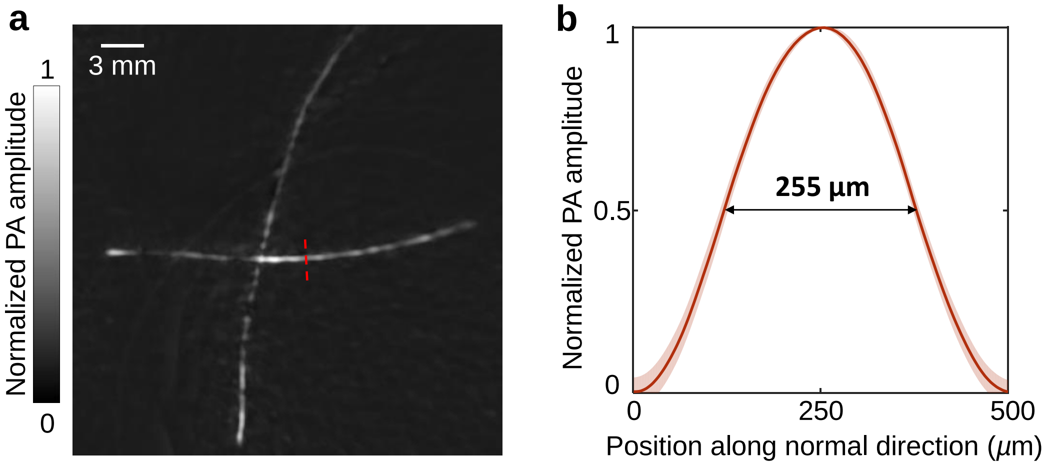

Supplementary Figure 4. Quantification of the in-plane resolution of the SBH-PACT system. **a,** A maximum amplitude projection (MAP) image of two crossed tungsten wires, each with a nominal diameter of 13 µm. **b,** The photoacoustic amplitude distribution along the red dashed line in (a). The in-plane resolution, defined as the FWHM of the amplitude distribution, is 255 µm.

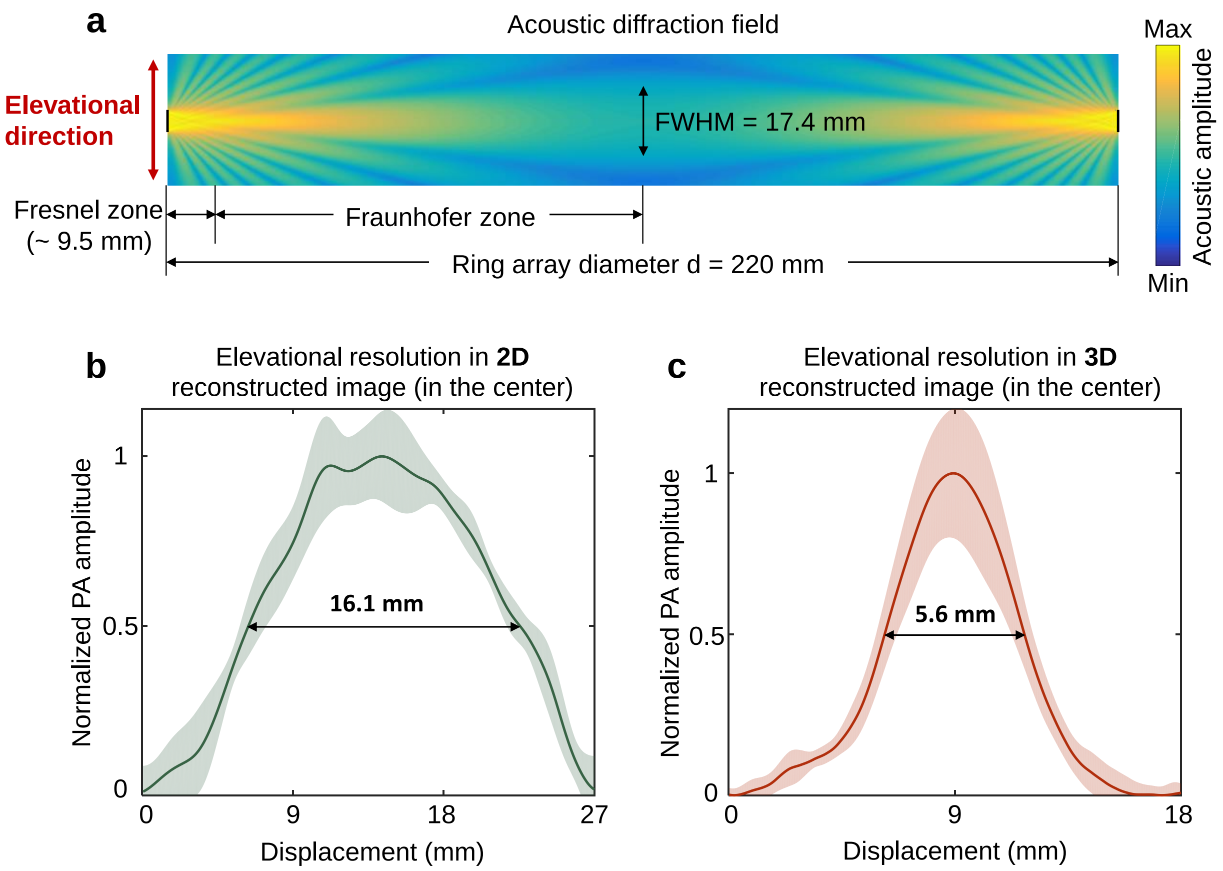

Supplementary Figure 5. Quantification of elevational resolution. **a,** Simulated acoustic diffraction field in the elevational direction. **b,** Line profile in the elevational direction of a carbon particle (20–50 μm, placed at the ring center) reconstructed by 2D back-projection. **c,** Line profile in the elevational direction of the same carbon particle reconstructed by 3D back-projection.

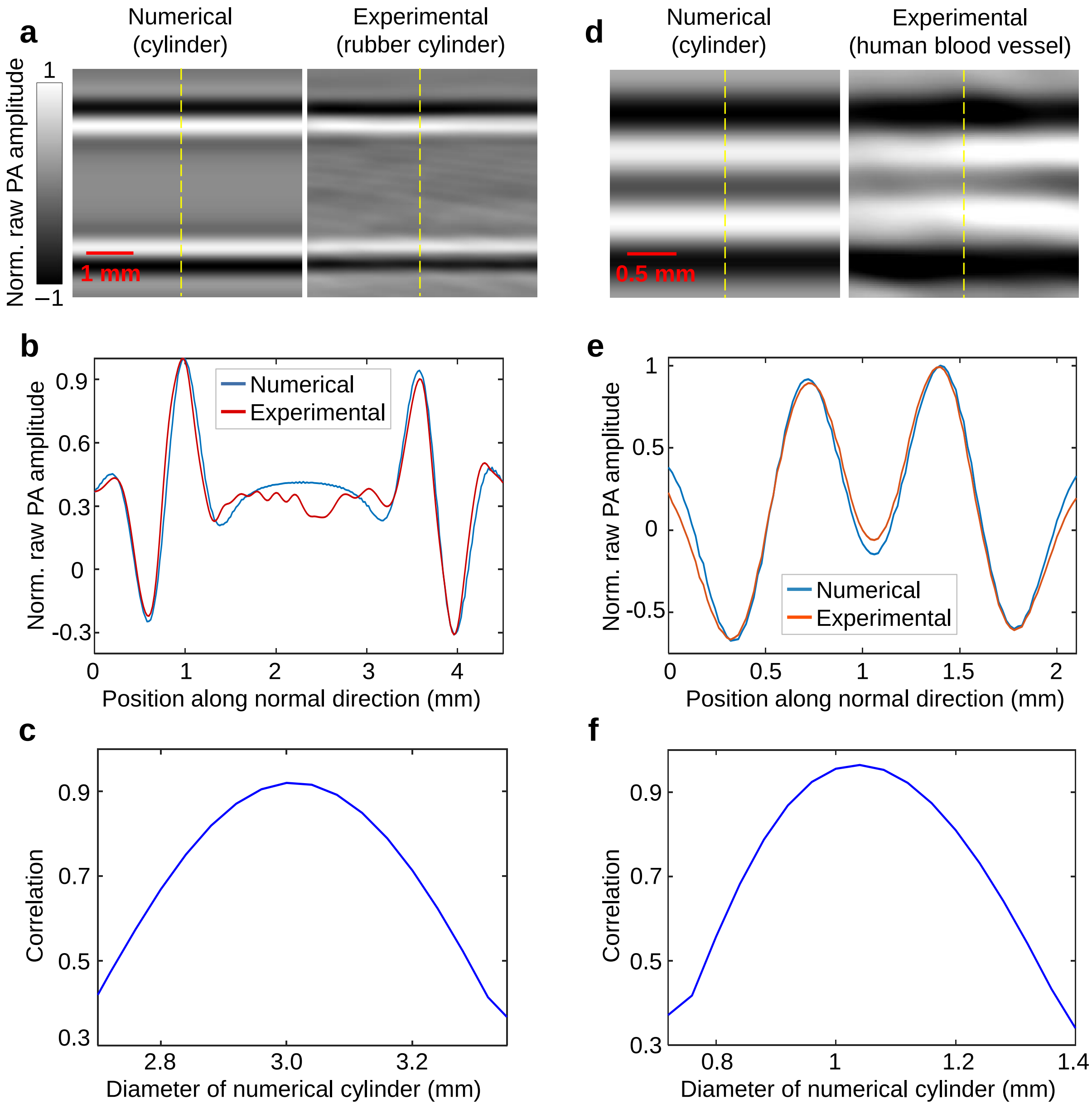

Supplementary Figure 6. Vascular diameter quantification. **a,** (left) Numerically simulated image of a cylinder with a diameter of 3 mm. (right) Experimental image of a rubber cylinder with a pre-known diameter of 3 mm. **b,** Photoacoustic amplitude distributions along the normal directions (yellow dashed lines) of the numerical cylinder and the rubber cylinder. **c,** Correlation coefficients between numerical cylinders with different diameters and the rubber cylinder. **d,** (left) Numerically simulated image of a cylinder with a diameter of 1.04 mm. (right) *In vivo* image of a section of a human blood vessel. **e,** Photoacoustic amplitude distributions along the normal directions (yellow dashed lines) of the numerical cylinder and the blood vessel. **c,** Correlation coefficients between numerical cylinders with different diameters and the blood vessel.

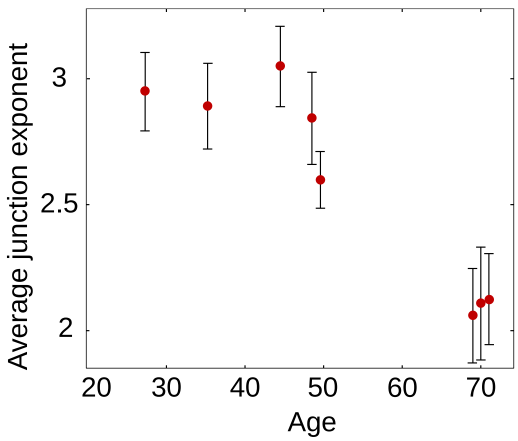

Supplementary Figure 7. The average junction exponents of the eight subjects, including the healthy volunteer and patients. Subjects' ages ranged from 27 to 71.

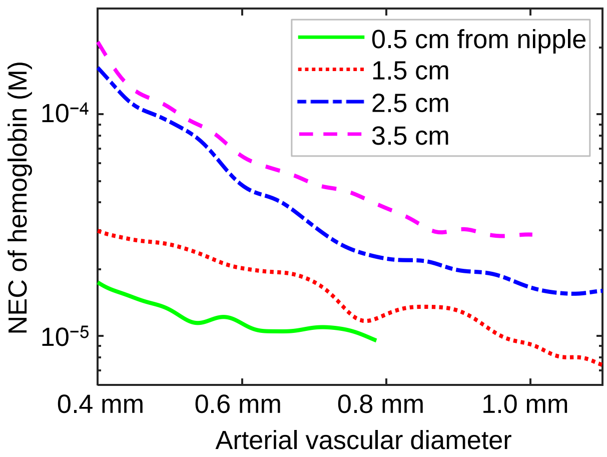

Supplementary Figure 8. The noise-equivalent molar concentration (NEC) of hemoglobin was used to quantify the detection sensitivity[2]. NEC values are plotted for arterial vessels with different diameters at different depths. The breast size was C cup and the incident fluence of SBH-PACT was approximately 20 mJ cm^{-2}.

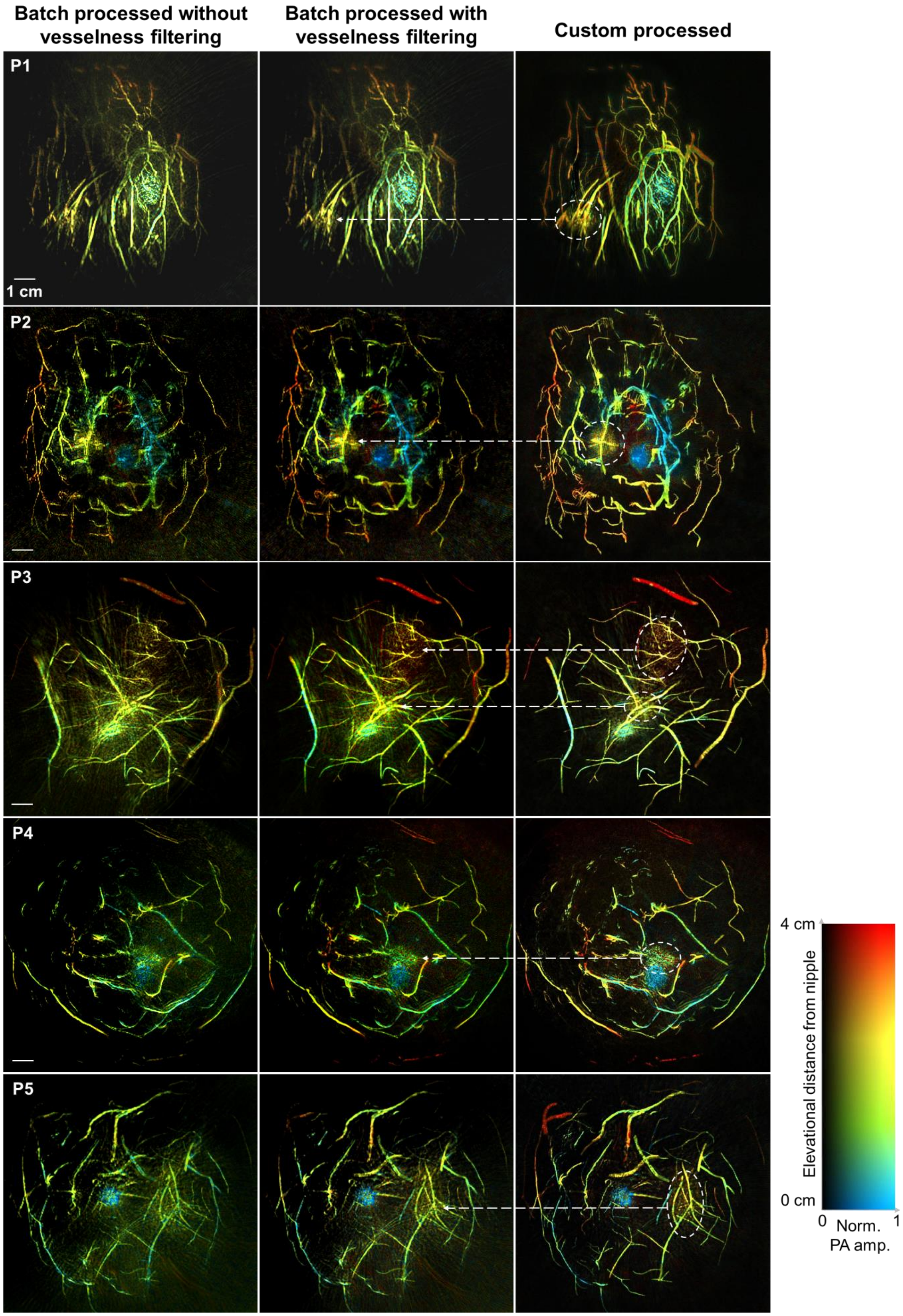
Batch processed without vesselness filtering
Batch processed with vesselness filtering
Custom processed
P1
1 cm
P2
P3
P4
P5
4 cm
0 cm
Elevational distance from nipple
0
Norm. PA amp.
1

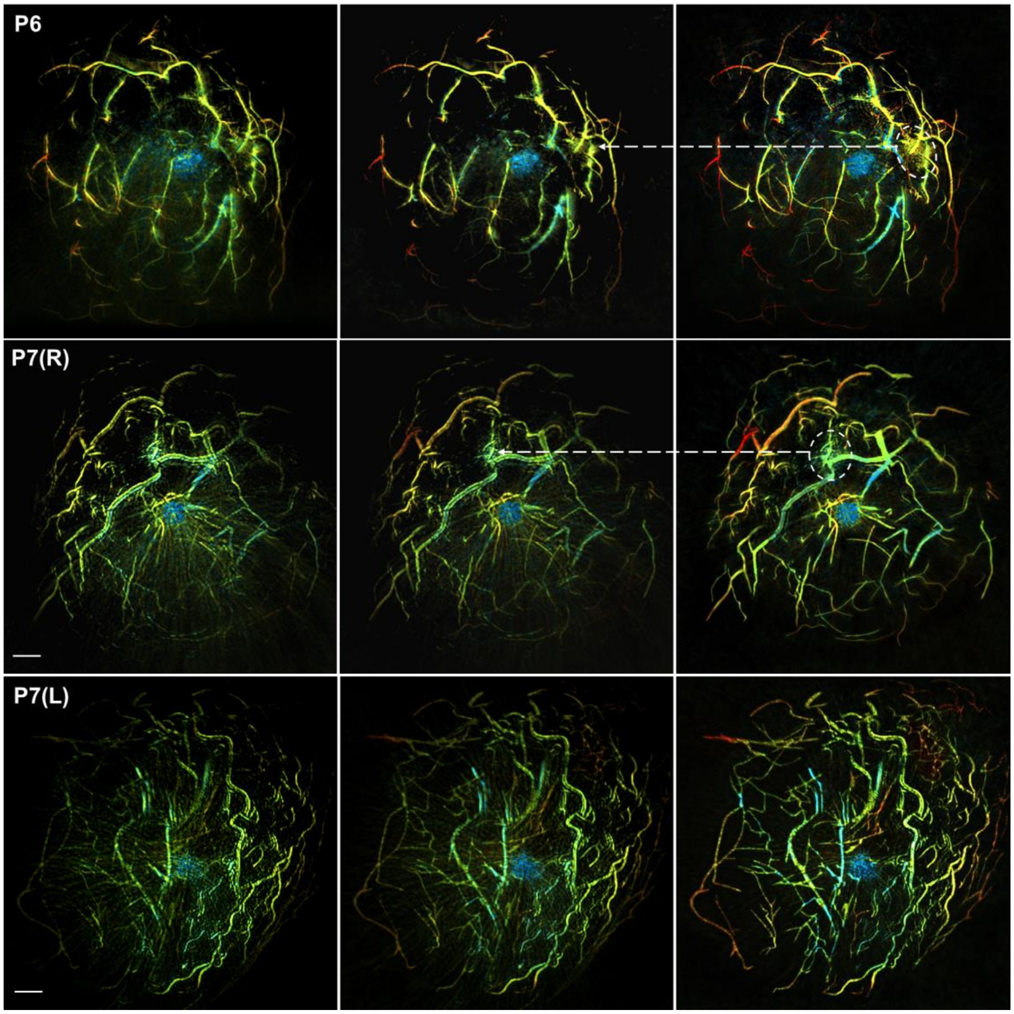

Supplementary Figure 9. Side-by-side comparisons of the depth-encoded angiograms that were batch processed without vesselness filtering, batch processed with vesselness filtering, and custom processed, respectively. All the tumors that are enclosed by white dashed circles can be visualized in the batch-processed images. The tumor in P7L is invisible in the angiograms although it is visible in the photoacoustic elastogram (Fig. 3e).

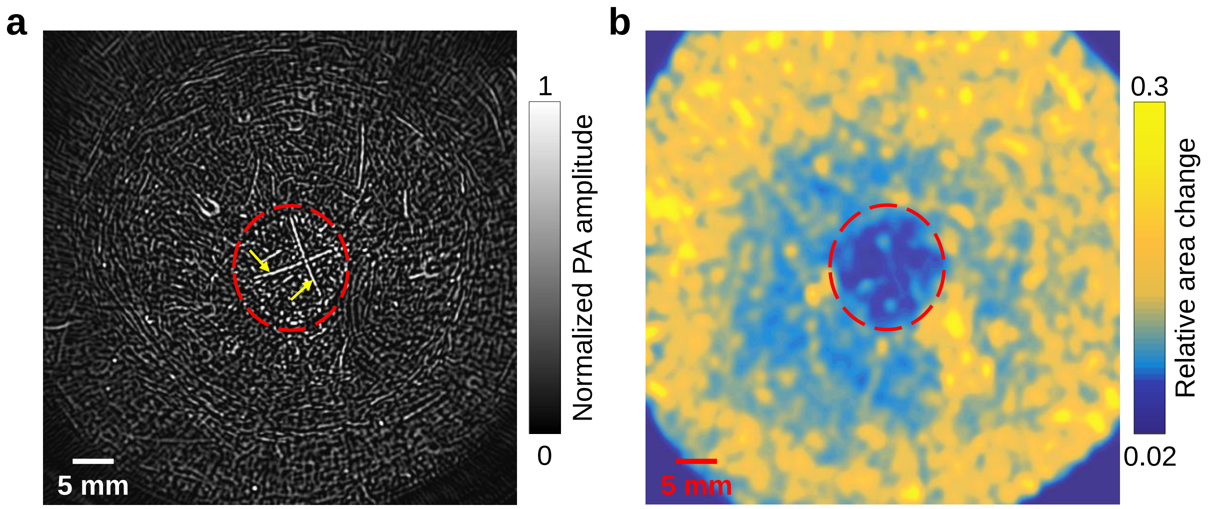

Supplementary Figure 10. SBH-PACT elastography of a breast mimicking phantom. **a,** Cross-sectional image of the phantom acquired by SBH-PACT. Hundreds of chopped human hairs were uniformly distributed in the phantom to mimic small blood vessels. To mark the location for comparison, two crossed tungsten wires (indicated by yellow arrows) were placed inside the ball (enclosed by the red dashed circle), which had a higher agar concentration to mimic a breast tumor. **b,** SBH-PACT elastographic image of the same cross section. Identified by the red dashed circle, the location of the agar ball is revealed correctly.

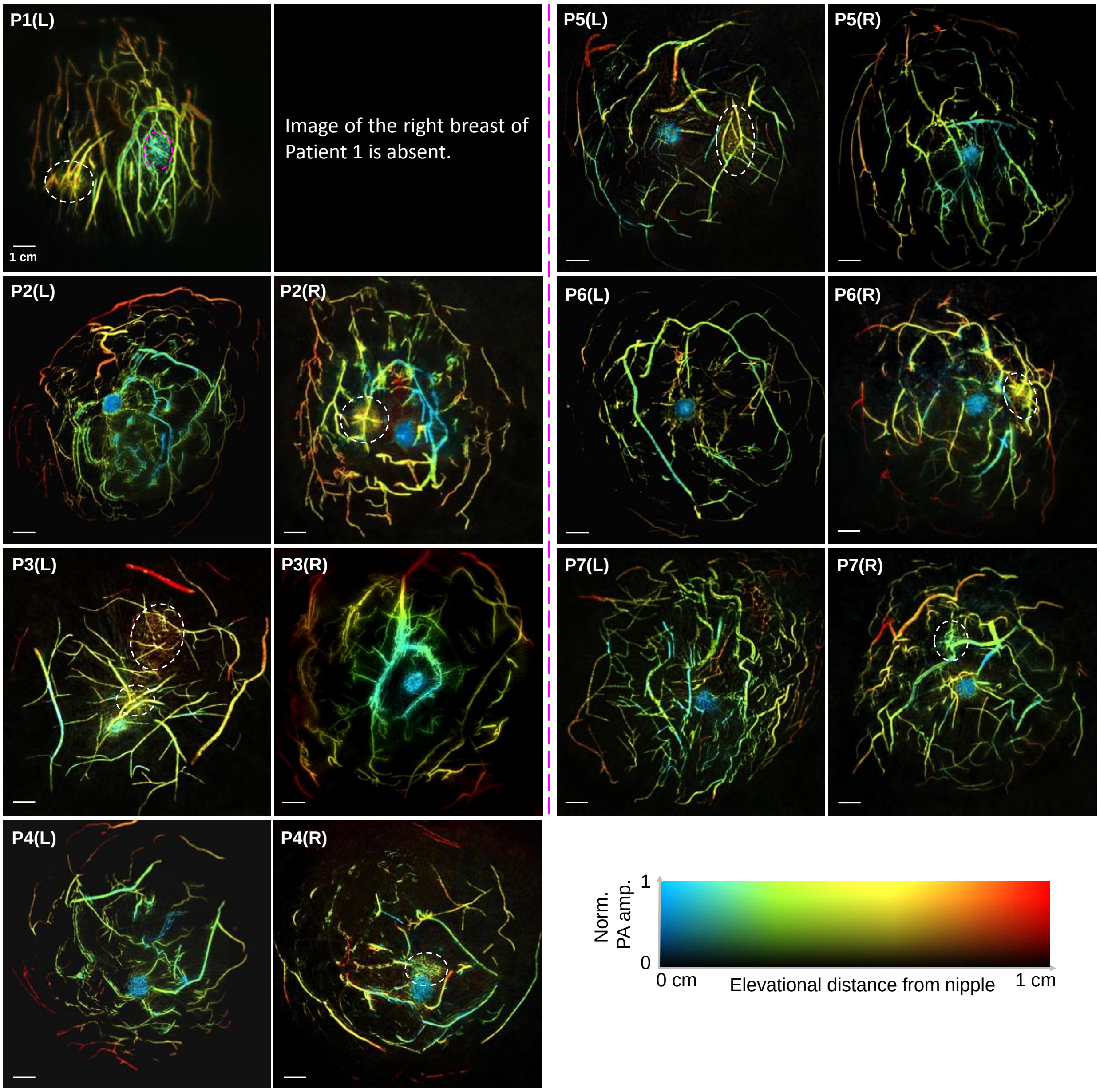

Supplementary Figure 11. Side-by-side comparisons between the left and right breast images of each patient. Visible tumors are enclosed by white dashed circles. The absence of the right breast image of Patient 1 was due to the lack of experience of the study coordinator, who did not check the breast position before imaging.

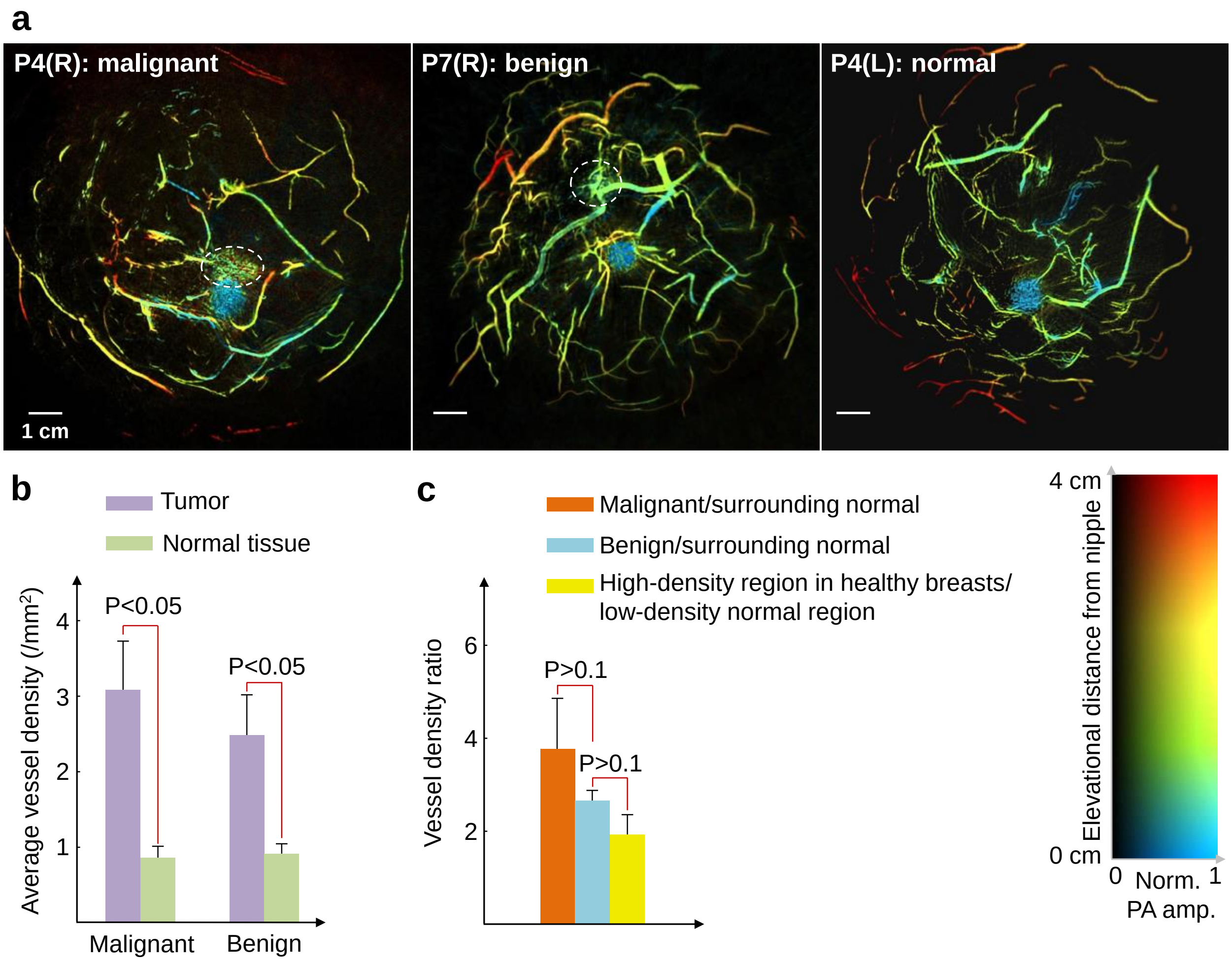

Supplementary Figure 12. Comparison of malignant, benign, and healthy cases. **a,** SBH-PACT images of breasts with a malignant tumor (P4(R)), a benign tumor (P7(R)), and no tumor (P4(L)). Breast tumors are enclosed with white dashed circles. **b,** Average vessel densities of tumors and surrounding normal tissues. **c,** The average vessel density ratio between the tumor and the normal tissue of malignant tumors is approximately 1.4 times higher than that of benign ones.

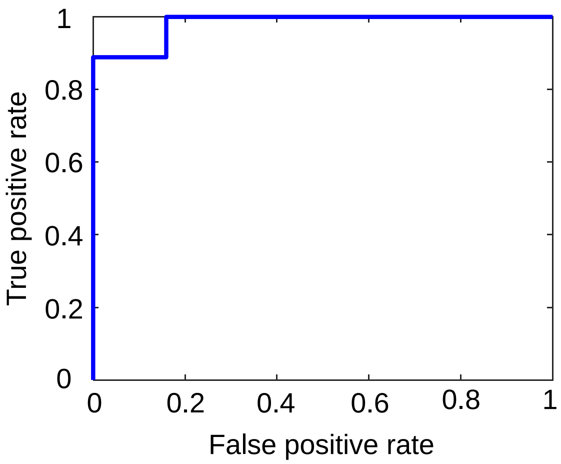

Supplementary Figure 13. The receiver operating characteristic (ROC) curve of tumor identification based on the sizes of the contiguous high vessel density regions. A threshold of numbers of pixels within (1855, 6379) produced a sensitivity of 89% and a specificity of 100%.

Supplementary Table

Supplementary Table 1. Sensitivities and specificities of tumor detection based on vessel-density thresholds obtained from the training data sets.

Trial	Sensitivity	Specificity
1	75%	100%
2	100%	50%
3	80%	100%
4	75%	100%
5	80%	100%
6	100%	67%
7	100%	67%
8	80%	100%
9	100%	75%
10	80%	100%
Average	87.0%	85.9%

Supplementary References

1. Jacques, S. L. Optical properties of biological tissues: a review. *Phys. Med. Biol*. **58**, 5007–5008 (2013).
2. Yao, J. & Wang, L. V. Sensitivity of photoacoustic microscopy. *Photoacoustics* **2**, 87–101 (2014).
