# Supplementary material for: Single-breath-hold photoacoustic computed tomography of the breast
Source: Nat Commun. 2018 Jun 15;9:2352. doi: 10.1038/s41467-018-04576-z (PMC6003984; doi:10.1038/s41467-018-04576-z)
Supplement: Supplementary file 2 — Description of Additional Supplementary Files [file 41467_2018_4576_MOESM2_ESM.pdf]

Description of Additional Supplementary Files

Supplementary Movie 1. Volumetric image of a phantom, which had an absorption coefficient of 0.05 cm^{-1} and a reduced scattering coefficient of 7 cm^{-1} to mimic the bulk optical properties of human breast tissue at 1064 nm wavelength. Three human hair fibers were embedded in the phantom. The total imaging depth along the elevational direction (*z*-axis) was ~5.2 cm. Tick mark: 1 cm.

Supplementary Movie 2. *In vivo* photoacoustic computed tomography of a healthy breast, visualizing the angiographic anatomy from the nipple to the chest wall. The entire volumetric image was acquired within a single breath hold (~15 sec).

Supplementary Movie 3. Arterial pulsatile deformation inside a breast. In the right panel, the normalized pixel value fluctuation in one artery and one vein are co-plotted.

Supplementary Movie 4. Photoacoustic computed tomography of a breast cross section showing minor deformations caused by breathing. The right panel is a co-plot of the relative area changes in selected triangular grids (red, tumor; blue, normal tissue).
